# Notes and Comments

**Published:** 1900-09

**Authors:** 


### Notes and Comments.[1]

The Dental Protective Association.—From letters and circulars sent out and published in the journals, the idea has become almost universal that the doors of the Protective Association were closed and no one could join that body hereafter. Regarding this, Dr. Crouse, in *Dental Digest,* says, "Nothing could be further from our plans. *The doors were closed only for defence against suits on the present bridge patents,* and this action has no bearing on the Association taking in new members and guaranteeing to protect them against the abuse of other illegal patent companies."

---

Comments on the Second Application of Arsenic for Devitalization of Pulp.—No. 1. A second application of arsenic is a great mistake. If the tissue within the pulp-chamber is found devitalized, there is no question concerning that within the root-canals. The sensitive point encountered at the apex is not vital pulp-tissue, but is due to an inflamed, irritable condition of the corpuscles of the cementum, from too large an amount of the arsenic left too long in the tooth, and requires the application of dialyzed iron to neutralize the arsenic, followed by soothing anodynes.—W. C. Barrett, in *Dental Brief.*

No. 2. The above vicious paragraph, having appeared in several publications, needs correction. It is equivalent to saying that destruction of the particle means death of the mass, or that an eschar on the finger is loss of vitality in the arm. It would be somewhat curious to note the mental aberration by which a man arrives at the conclusion that a broach in a nerve-canal, surrounded by dentine, irritates a corpuscle in the cementum, and especially how that cement corpuscle, devoid of nerve-structure, responds with such suddenness to a prick in nerve-tissue not at all connected with it.—Charles L. Hungerford, in *Western Dental Journal.*

---

[1] The assistant editor solicits contributions for this department,—new methods, new remedies and formulas, or any short practical note which may prove of value to the practitioner or student. Address 1338 Walnut Street, Philadelphia.

A Remarkable Reflex.—I was called at one A.M. to see a young man twenty-four years old, who, the message said, was dying. When I reached the house, he was trying to lie on a couch, had a flushed face and congested eyes, perspiring profusely, unable to remain in any position for more than a minute at a time, short spasmodic attempts to breathe, and complaining solely of constricted pain over the heart, over which he kept his hand constantly. Could obtain no history of the case from him, but from family learned that he had been taken very suddenly, in the manner above described, about two hours before. Had had toothache in lower left molar for about a week, and had used camphor freely to put on gum, and more freely than usual just before attack came on.

Gave him aconite every fifteen minutes, and within one hour he was resting quietly. Saw him the following two days, feeling quite well with the exception of some toothache, but with no more chest pain. Was called again the second night, and found him worse than the first time. I again gave him aconite, which seemed to give a slight relief, but finally was compelled to give him an opiate, as it took two men to restrain him in bed.

I now knew that the trouble must come from his tooth, for when his tooth ached he had no other pains, and when he had chest pain his tooth felt easy. The next day I took a dentist with me, who pulled the tooth, which was an unusual one, having exostosed roots, that had extended quite deeply into the bone. His recovery was rapid after the extraction.

I give this as an unusual case of reflex pain, and with the hope that it may show that reflex pains are more frequent than we imagine.—Charles B. Kern, M.D., in *Medical Visitor.*

The editor of the *Dominion Dental Journal* says of the above report, "It is a choice sample of medical ignorance of dental subjects. The 'Remarkable Reflex' was a simple case which the average dental student would have diagnosed and treated directly, without any of the preliminary humbug which the wiseacre had to use before he made the remarkable discovery that it was toothache."

